# Neurofibromin 1 Impairs Natural Killer T-Cell-Dependent Antitumor Immunity against a T-Cell Lymphoma

**DOI:** 10.3389/fimmu.2017.01901

**Published:** 2018-01-05

**Authors:** Jianyun Liu, Richard M. Gallo, Masood A. Khan, Gourapura J. Renukaradhya, Randy R. Brutkiewicz

**Affiliations:** ^1^Department of Microbiology and Immunology, Indiana University School of Medicine, Indianapolis, IN, United States; ^2^College of Applied Medical Sciences, Al-Qassim University, Buraidah, Saudi Arabia; ^3^Food Animal Health Research Program (FAHRP), Ohio Agricultural Research and Development Center (OARDC), Department of Veterinary Preventive Medicine, The Ohio State University, Wooster, OH, United States

**Keywords:** neurofibromin 1, CD1d, natural killer T cells, T-cell lymphoma, antitumor immunity

## Abstract

Neurofibromin 1 (NF1) is a tumor suppressor gene encoding a Ras GTPase that negatively regulates Ras signaling pathways. Mutations in NF1 are linked to neurofibromatosis type 1, juvenile myelomonocytic leukemia and Watson syndrome. In terms of antitumor immunity, CD1d-dependent natural killer T (NKT) cells play an important role in the innate antitumor immune response. Generally, Type-I NKT cells protect (and Type-II NKT cells impair) host antitumor immunity. We have previously shown that CD1d-mediated antigen presentation to NKT cells is regulated by cell signaling pathways. To study whether a haploinsufficiency in NF1 would affect CD1d-dependent activation of NKT cells, we analyzed the NKT-cell population as well as the functional expression of CD1d in *Nf1*^+/−^ mice. *Nf1*^+/−^ mice were found to have similar levels of NKT cells as wildtype (WT) littermates. Interestingly, however, reduced CD1d expression was observed in *Nf1*^+/−^ mice compared with their WT littermates. When inoculated with a T-cell lymphoma *in vivo, Nf1*^+/−^ mice survived longer than their WT littermates. Furthermore, blocking CD1d *in vivo* significantly enhanced antitumor activity in WT, but not in *Nf1*^+/−^ mice. In contrast, a deficiency in Type-I NKT cells increased antitumor activity in *Nf1*^+/−^ mice, but not in WT littermates. Therefore, these data suggest that normal NF1 expression impairs CD1d-mediated NKT-cell activation and antitumor activity against a T-cell lymphoma.

## Introduction

Neurofibromatosis type 1 is an autosomal-dominant disorder caused by a mutation in a tumor suppressor gene encoding the protein neurofibromin 1 (NF1) (1), affecting 1 in 3,500 individuals worldwide. NF1 is a p21^ras^ (Ras) guanosine triphosphatase (GTP)-activating protein (GAP). It catalyzes the hydrolysis of Ras-GTP, thus negatively regulating multiple Ras-dependent cellular signaling pathways (1). Mutations in *NF1* are associated with many diseases, including hematopoietic cancers such as myeloid leukemia and diffuse plexiform neurofibromas (2). Extensive studies from human tissue analyses and mouse models have discovered that loss of heterogyzosity (LOH) of *NF1* in Schwann cells and a heterozygous *NF1* microenvironment are both important for the formation of neurofibromas (3, 4). LOH may also explain the localized formation of tumors in patients with neurofibromatosis type 1 (1).

Ras-dependent signaling pathways have been shown to be important for αβ T-cell positive selection (5). Because NF1 is a negative regulatory GAP and highly expressed in leukocytes (6), the absence of NF1 may affect T-cell development. An *Nf1^−/−^* mutation is embryonic lethal (1). Therefore, the method of *Nf1^−/−^* fetal liver reconstitution to immune-deficient mice, such as Rag1 KO mice, has been used to study T-cell development in the absence of NF1 (7). Although an *nf1* deficiency in mice increases T-cell numbers in both thymus and spleen, it also causes impaired proliferation of T cells in response to *in vitro* stimulation (7). Moreover, antigen receptor-induced proliferation is also defective in NF1-deficient peripheral B cells (8), implicating a positive (but unknown) role for NF1 in regulating B and T-cell receptor (TCR)-induced proliferation. An earlier study indicated that NF1 promotes thymocyte positive selection, but has no effect on negative selection (9). Increasing evidence also suggests that NF1 may function in other cellular processes besides negatively regulating Ras function (10). For example, the Sec14-homology domain of NF1 is involved in forming a bipartite lipid-binding module, and possibly binds to cellular glycerophospholipid ligands (11). The loss of NF1 in *Drosophila* causes a reduction in body size, which is rescued by increasing cAMP protein kinase (PKA) signaling; this suggests that NF1 may also regulate the cAMP signaling pathway in a GAP-independent manner (12).

Natural killer T (NKT) cells express both natural killer (NK) and T-cell markers. Unlike conventional T cells which recognize peptide antigens presented by MHC class I and II molecules, NKT cells are activated by lipid antigens presented by the MHC class I-like molecule, CD1d. CD1d-deficient mice lack NKT cells and NKT-cell development requires positive selection in the thymus, similar to conventional T-cell development (13). Ras/mitogen-activated protein kinase (MAPK) signaling pathways, which are important for αβ T-cell positive selection (5), have also been shown to be critical for NKT-cell development (14). Furthermore, previous work from our laboratory has demonstrated that stimulation of MAPK pathways affects CD1d-mediated antigen presentation (15, 16). We have found that activation of the p38 pathway inhibits, whereas activation of ERK pathway increases, CD1d-mediated antigen presentation to NKT cells, likely through regulating the trafficking of CD1d molecules in antigen-presenting cells (15). In line with this, we reported that anthrax toxin inhibits CD1d-mediated antigen presentation by targeting the ERK pathway (16).

Based on TCR usage, NKT cells can be divided into two groups: Type-I (invariant) and Type-II (other CD1d-restricted) NKT cells. Type-I NKT (also called *i*NKT) cells express an invariant TCR α-chain rearrangement (Vα14Jα18 in mice and Vα24Jα18 in humans) that is associated with β-chains of limited diversity (Vβ8.2, Vβ7, and Vβ2 in mice; Vβ11 in humans). The glycolipid α-galactosylceramide (α-GalCer or PBS57), originally derived from a marine sponge, has been shown to be a specific activator of *i*NKT cells in a CD1d-dependent manner (17, 18). Type-II NKT cells are less well-defined, due to a paucity of ligands identified that are recognized by these NKT cells (19, 20). However, by studying CD1d-deficient (lacking both Type-I and Type-II NKT cells) and Jα18-deficient mice (lacking only Type-I NKT cells), it is believed that Type-II NKT cells are similar to T regulatory cells (Tregs) and are mostly immunosuppressive (21). In line with this idea, Type-II NKT cells have been shown to impair tumor immunosurveillance in a CD1d-dependent manner (22).

In the current study, we asked whether NF1, a negative regulator of Ras/MAPK pathways, impacts CD1d-dependent antitumor activity by NKT cells. Because an *Nf1^−/−^* mutation is embryonic lethal, a haploinsufficient (*Nf1*^+/−^) mouse model is commonly used for the study of NF1 function *in vivo*. We analyzed NKT-cell activity as well as the functional expression of CD1d in *Nf1*^+/−^ mice, in order to determine whether a haploinsufficiency in NF1 would affect the CD1d/NKT-cell axis in the context of NKT-cell-mediated antitumor activity.

## Materials and Methods

### Animals

Female C57BL/6 wildtype (WT) mice were obtained from The Jackson Laboratory (Bar Harbor, ME, USA). Male *Nf1*^+/−^ mice were kindly provided by Dr. Wade Clapp (Indiana University, Indianapolis, IN, USA). *CD1d1* KO (*CD1d1^−/−^*) mice on the C57BL/6 background (23) were a kind gift from Dr. Luc Van Kaer (Vanderbilt University, Nashville, TN, USA). Jα18-deficient C57BL/6 mice were also obtained from Dr. Van Kaer, with permission from Professor M. Taniguchi (Chiba University, Chiba, Japan). All mice were bred in specific pathogen-free facilities at the Indiana University School of Medicine. *Nf1*^+/−^ mice were backcrossed to *CD1d1^−/−^* mice or *J*α*18^−/−^* to obtain *Nf1*^+/−^/*CD1d1^−/−^* and *Nf1*^+/−^/*J*α*18^−/−^* mice, respectively. All mice were age- and sex-matched littermates, both males and females were utilized, and used in all experiments between 8 and 16 weeks of age. All animal procedures were approved by the Indiana University School of Medicine’s Institutional Animal Care and Use Committee.

### Cell Lines

The Tap 2-deficient RMA/S T-cell lymphoma cell line was kindly provided by Drs. J. Yewdell and J. Bennink (National Institutes of Health, Bethesda, MD, USA). These cells were transfected with the pcDNA3.1-neo vector alone (RMA/S-V) or the vector with a mouse *cd1d1* cDNA insert (RMA/S-CD1d) as previously described (23). MC57G–CD1d cells were generated by transfecting the methylcholanthrene-induced fibrosarcoma cell line MC57G with a pSRα vector encoding mouse *cd1d1* cDNA (a kind gift from Dr. S. Balk, Harvard University, Cambridge, MA, USA).

### Antibodies and Reagents

Allophycocyanin (APC)-conjugated, PBS57-loaded, and unloaded CD1d tetramers were provided by the NIH Tetramer Core Facility (Atlanta, GA, USA). APC-, Phycoerythrin (PE)-, and fluorescein isothiocyanate (FITC)-conjugated monoclonal antibodies (mAb) against murine NK cell-, B-cell- or T-cell-specific markers, including NK1.1, MHC class II, CD11c, B220, CD1d (1B1), CD4, CD8, and TCRβ, were purchased from BD Biosciences (San Diego, CA, USA). PE/Cy7-conjugated anti-CD21 and PerCP/Cy5.5-conjugated anti-CD23 were from Biolegend (San Diego, CA, USA). The mouse CD1d-specific mAb 1H6 generated by our laboratory has been previously described (24). The isotype control mAb TW2.3 was kindly provided by Drs. J. Yewdell and J. Bennink (NIH, Bethesda, MD, USA). 1H6 and TW2.3 hybridoma supernatants were purified by immobilized protein A agarose beads for *in vivo* use.

### Flow Cytometry

Thymocytes and splenocytes were harvested using standard procedures. Liver mononuclear cells (LMNCs) were harvested as described previously (25). To obtain bone marrow-derived dendritic cells (BMDCs), bone marrow cells obtained from mouse femurs and tibias were cultured in the presence of IL-4 (10 ng/mL) and GM-CSF (10 ng/mL) for 7 days. For flow cytometry analyses, single-cell suspensions of all indicated cell types were prepared, and 1 × 10^6^ cells were incubated at 4°C for 30 min with various mAb as indicated. The cells were washed three times with HBSS containing 0.1% bovine serum albumin (BSA; Sigma-Aldrich, St. Louis, MO, USA). All cells were fixed with 1% paraformaldehyde in PBS and analyzed on a FACSCalibur or LSRII (Becton Dickinson, San Jose, CA, USA).

### T-Cell Stimulation Assays

Bone marrow-derived dendritic cells from *Nf1*^+/−^ mice and their littermates were incubated with the mouse Type-I NKT hybridoma N38-2C12 (26) or Type-II NKT hybridoma N37-1A12 (27) [both hybridomas kindly provided by Dr. K Hayakawa (Fox Chase Cancer Center, Philadelphia, PA, USA)]. 5 × 10^4^ hybridoma cells and 5 × 10^5^ BMDCs were added to triplicate wells in 96-well microtiter plates for 24 h. Secreted IL-2 levels in the supernatants were measured by ELISA.

### Western Blot Analysis

Thymocytes and splenocytes were lysed, separated on a 10% SDS-PAGE gel and then transferred to a polyvinylidene difluoride (PVDF) membrane (Merck Millipore, Billerica, MA, USA). The blot was then probed with phospho-JNK1/2 or ERK1/2-specific antibodies (Cell Signaling Technology, Inc., Danvers, MA, USA), and developed using chemiluminescence prior to exposure on film. The same membrane was then stripped and reprobed with total JNK1/2- or ERK1/2-specific antibodies (Cell Signaling Technology Inc.). Images were quantified using ImageJ (1.37v; National Institutes of Health, Bethesda, MD, USA).

### *In Vitro* Stimulation of NKT Cells

Liver mononuclear cells (2.5 × 10^5^ cells/well) from *Nf1*^+/−^ mice or WT littermates were cocultured with α-GalCer-pulsed MC57G–CD1d cells (5 × 10^5^ cells/well) in triplicate wells of a 96-well microtiter plate. After culture at 37°C for 48 h, the supernatants were collected for the analysis of NKT-cell production of IFN-γ, IL-4, and IL-13 by ELISA.

### Tumor Inoculation

*Nf1*^+/−^, *CD1d1^−/−^, Nf1*^+/−^/*CD1d1^−/−^, J*α*18^−/−^, Nf1*^+/−^/*J*α*18^−/−^*, and their WT littermates were inoculated intraperitoneally (i.p.) with 5 × 10^5^ RMA/S-V or RMA/S-CD1d cells in 500-µL IMDM media supplemented with 5% FBS. The mice were monitored for up to 60 days posttumor inoculation, as previously described (23). To block CD1d *in vivo*, the mice were injected i.p. with 50 μg/mouse of purified mouse CD1d-specific antibody (1H6) or isotype control mAb in PBS on days 1, 5, 10, and 20 posttumor inoculation.

### Statistics

Graphs were generated and statistics calculated using GraphPad Prism 6 (GraphPad Software, La Jolla, CA, USA). The mean of triplicates of a representative assay is shown with error bars representing the SEM, using Student’s *t*-test analyses. For the statistical analysis of survival rate, the log-rank test was performed. A *p*-value < 0.05 was considered significant.

## Results

### Increased JNK and ERK Activation in *Nf1*^+/−^ Mice

**Figure 1:**
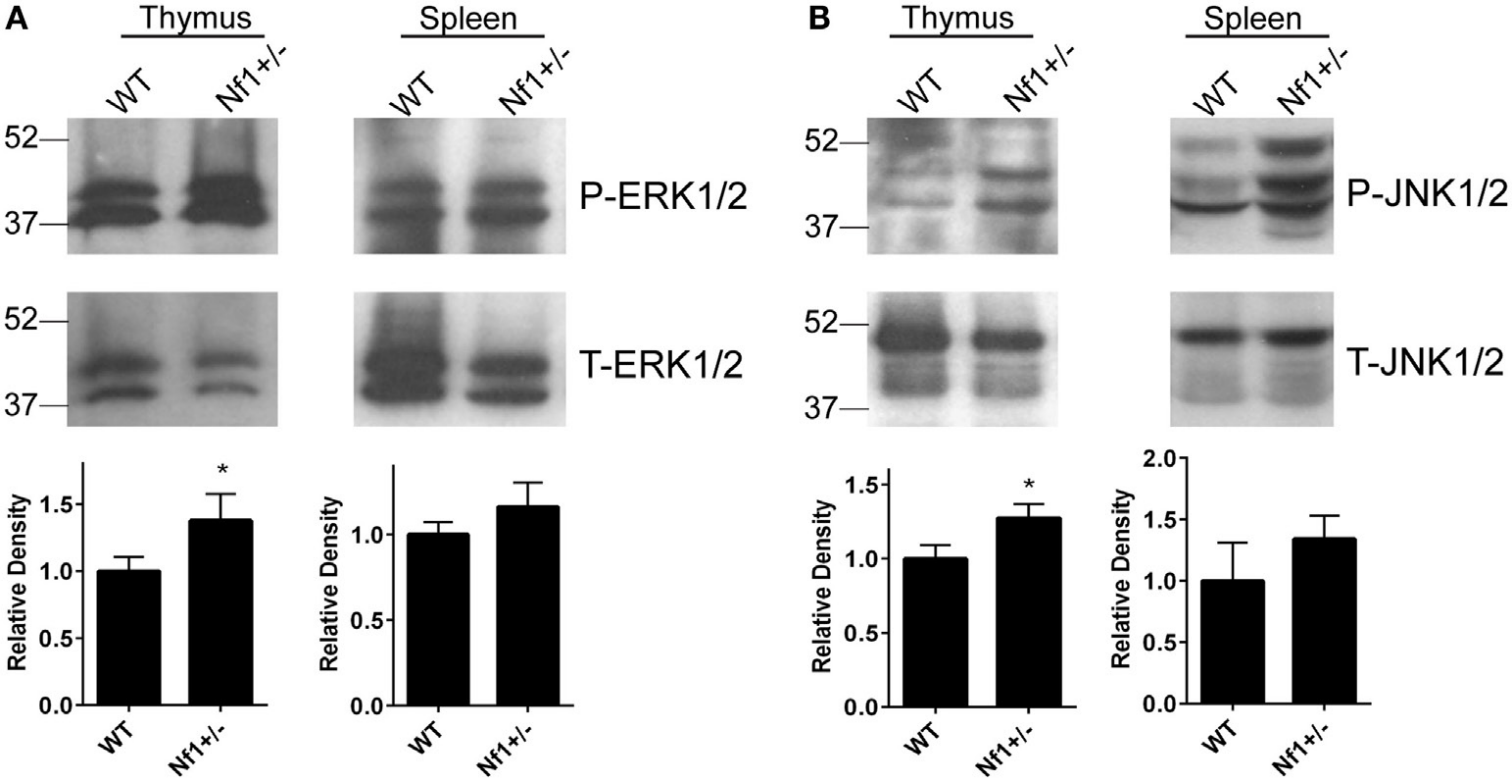
Increased activation of ERK and JNK in the spleen and thymus of *Nf1*^+/−^ mice. Splenocytes and thymocytes were treated with Phorbol 12-myristate 13-acetate (100 ng/mL) for 30 min. The cells were then lysed and resolved on a 10% SDS-PAGE gel for the detection of phosphorylated and total ERK1/2 **(A)** and JNK1/2 **(B)** expression by Western blot analysis. The relative levels of phosphorylated ERK1/2 and JNK1/2 compared with the total respective proteins were quantified by densitometry. Combined results from multiple experiments are shown in the bar graphs. The data are plotted as the mean ± SD. *p < 0.05.

Previous reports have shown increased Ras-GTP levels in unstimulated thymocytes from *Nf1*^+/−^ mice when compared with WT mice (7). In our study, we also observed elevated ERK phosphorylation in splenocytes and thymocytes when they were stimulated with Phorbol 12-myristate 13-acetate (Figure 1A; Figures S7A,B in Supplementary Material), suggesting elevated activation of the Ras/ERK pathway in *Nf1*^+/−^ mice. Compared with their WT littermates, *Nf1*^+/−^ mice were also found to have elevated JNK activation in the spleen and thymus (Figure 1B; Figures S7C,D in Supplementary Material). We did not observe any hyperactivation of p38 in the thymus or spleen from *Nf1*^+/−^ mice (data not shown). These data indicate that elevated Ras-GTP activity causes hyperactivation of the ERK and JNK pathways in *Nf1*^+/−^ mice.

### Comparable *i*NKT-Cell Population in WT and *Nf1*^+/−^ Mice

**Figure 2:**
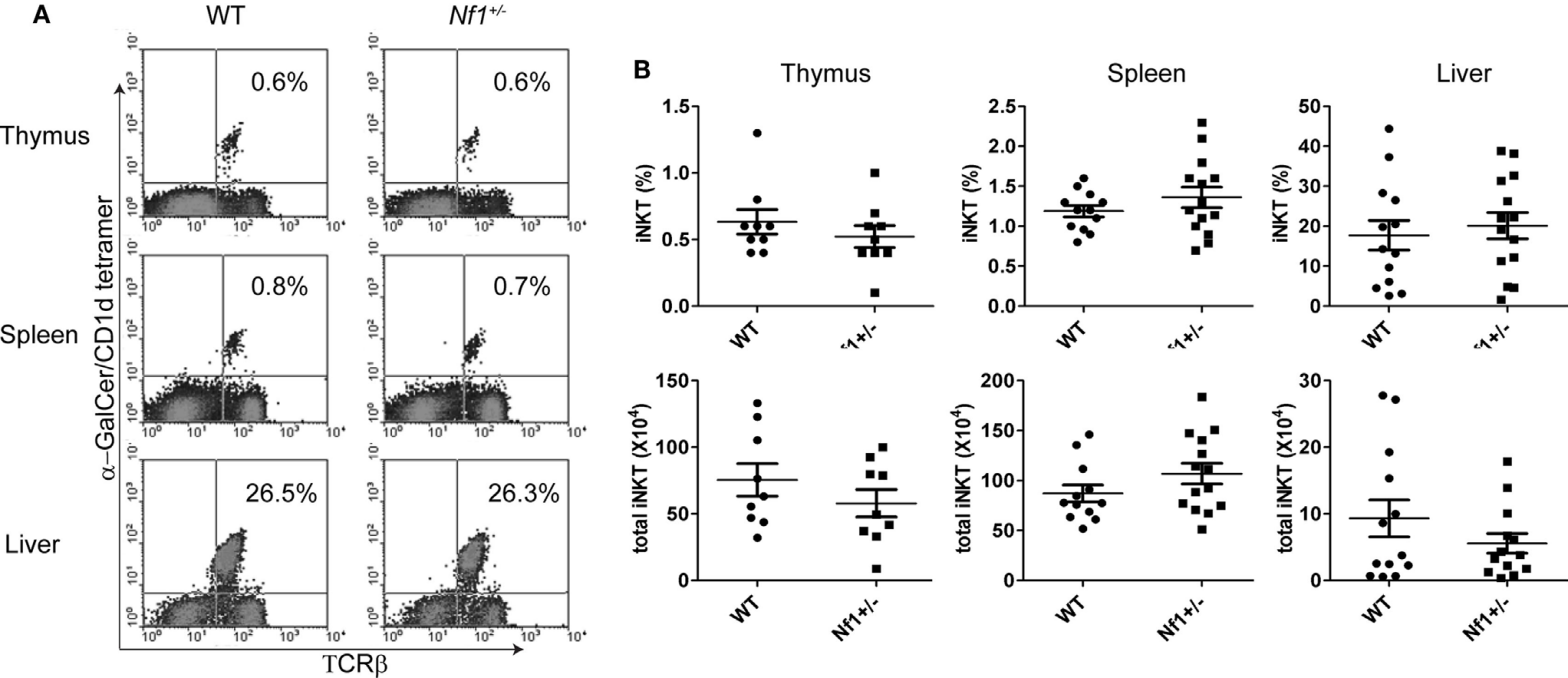
Comparable numbers of *i*NKT cells in wildtype (WT) and *Nf1*^+/−^ mice. **(A)** Thymocytes, splenocytes, and liver mononuclear cells from *Nf1*^+/−^ mice and WT littermates were stained with α-GalCer-loaded CD1d tetramers and a TCR-β-specific antibody for the identification of *i*NKT cells, identified in the upper right quadrant. **(B)** Percentages (upper) and total numbers (lower) of *i*NKT cells are summarized for the thymus, spleen and liver. Pooled data from three independent experiments are shown. Each dot represents an individual mouse. The data are plotted as mean ± SEM.

Previous studies have suggested that an NF1 deficiency increases the number of immature and mature conventional T cells *in vivo*, but reduces cell proliferation in response to TCR and IL-2 stimulation *in vitro* (7). NF1 promotes thymocyte positive selection, a process that is also required for NKT-cell development (9, 28). To determine if an NF1 deficiency affects *i*NKT-cell development, we compared the *i*NKT-cell populations in thymus, spleen, and liver from *Nf1*^+/−^ mice to those from WT littermates. We found there were comparable levels of *i*NKT cells in WT and *Nf1*^+/−^ mice (Figure 2), suggesting that a haploinsufficiency in NF1 has a minimal effect on NKT-cell development and their numbers in the periphery.

### Lower CD1d Expression on BMDCs from *Nf1*^+/−^ Mice

**Figure 3:**
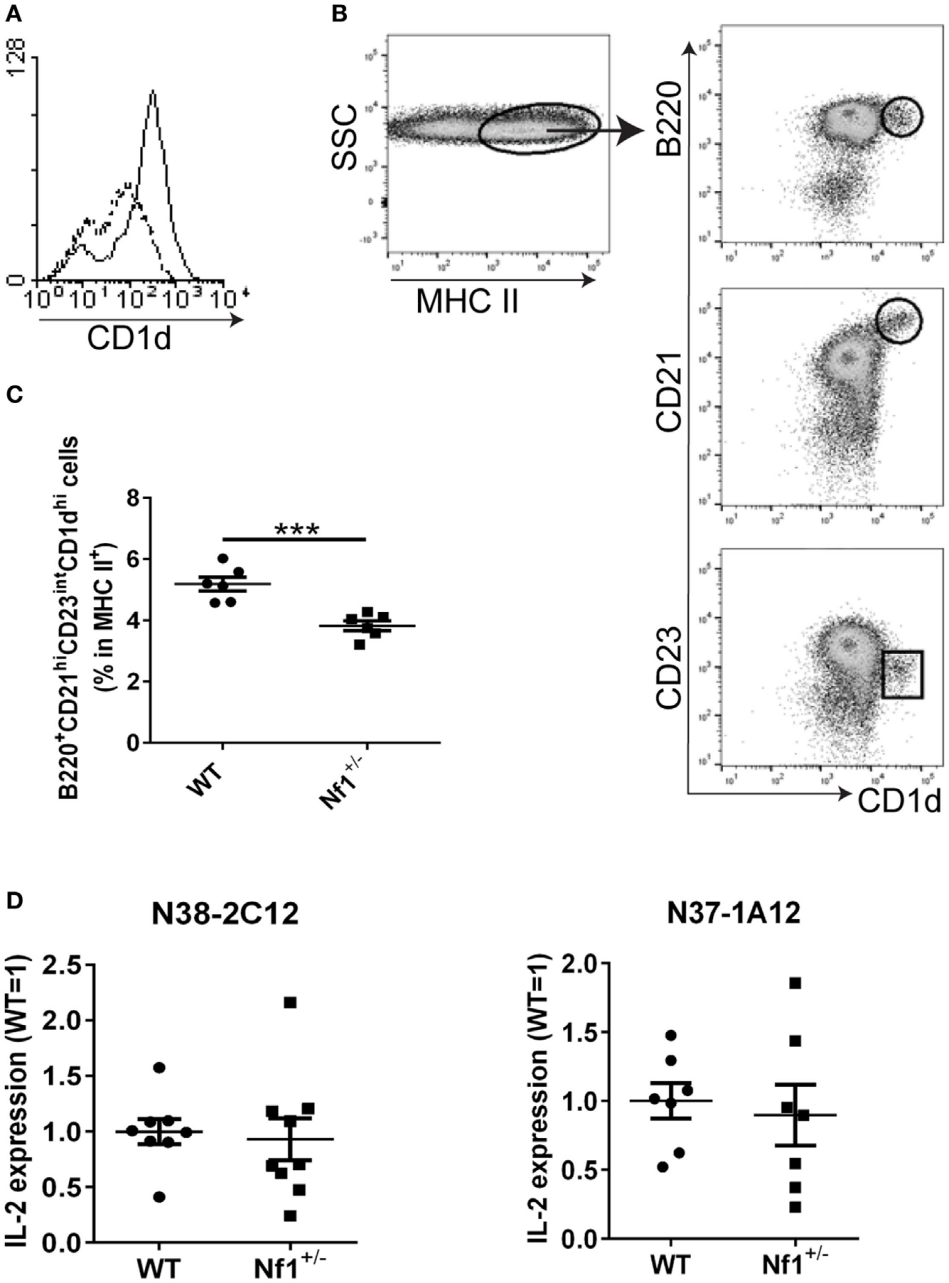
Lower CD1d expression on cells from *Nf1*^+/−^ mice. **(A)** Bone marrow-derived dendritic cells (BMDCs) from *Nf1*^+/−^ and wildtype (WT) mice were fixed and stained with the anti-CD1d mAb, 1B1. CD1d-specific staining from a representative *Nf1*^+/−^ mouse (dotted line) was overlaid with that of a WT littermate (solid line). **(B)** Splenocytes from *Nf1*^+/−^ mice or WT littermates were stained with MHC II-, B220-, CD21-, CD23-, and CD1d-specific antibodies. MHC II^+^ cells were gated and further analyzed for B220, CD21, CD23, and CD1d expression by flow cytometry. The circled population corresponds to B220^+^CD21^hi^CD23^int^CD1d^hi^ splenocytes. Combined results from multiple experiments are shown in **(C)**. Each dot represents an individual mouse. ***p < 0.001. **(D)** BMDCs from *Nf1*^+/−^ mice or WT littermates were cocultured with the NKT-cell hybridomas, N38-2C12 and N37-1A12. The activation of NKT cells by BMDCs was determined by ELISA, measuring IL-2 secretion in the supernatants. The relative levels of IL-2 production in *Nf1*^+/−^ BMDCs compared with WT (WT = 1) are indicated. Combined results from multiple experiments are shown. The data are plotted as the mean ± SEM. Each dot represents an individual mouse.

Although a haploinsufficiency in NF1 did not seem to affect *i*NKT-cell development, we found that BMDCs from *Nf1*^+/−^ mice expressed lower levels of CD1d (but similar amounts of MHC class I and II) on the cell surface compared with WT BMDCs (Figure 3A; Figure S1 in Supplementary Material). Furthermore, similar to the reduced CD1d expression observed in *Nf1*^+/−^ BMDCs, there was also a significant decrease in the splenic B220^+^CD21^hi^CD23^int^CD1d^hi^ population in *Nf1*^+/−^ mice (Figures 3B,C). These cells express a high level of CD21 and low to intermediate levels of CD23, suggesting they are marginal zone B (MZB) cells. We also analyzed DCs (MHC II^+^ CD11c^+^) and macrophages (MHC II^+^ F4/80^+^) for CD1d expression, but there were no differences between WT and *Nf1*^+/−^ mice (data not shown). Although BMDCs from *Nf1*^+/−^ mice expressed less CD1d on their surface, they were similar to WT BMDCs in their ability to activate both Type-I and Type-II NKT-cell hybridomas (Figure 3D). Interestingly, thymocytes from both WT and *Nf1*^+/−^ mice express similar levels of CD1d and have a comparable ability in stimulating NKT cells (Figure S8 in Supplementary Material); this suggests that a haploinsufficiency in NF1 does not alter the positive selection of NKT cells in the thymus. Overall, we conclude that a haploinsufficiency in NF1 reduces CD1d surface expression, but the decrease in CD1d expression in *Nf1*^+/−^ cells is likely still above the normal threshold level necessary to activate NKT cells. This may help explain why *Nf1*^+/−^ mice have a similar level of *i*NKT cells *in vivo* as their WT littermates.

### Increased Activation of *i*NKT Cells from *Nf1*^+/−^ LMNCs

**Figure 4:**
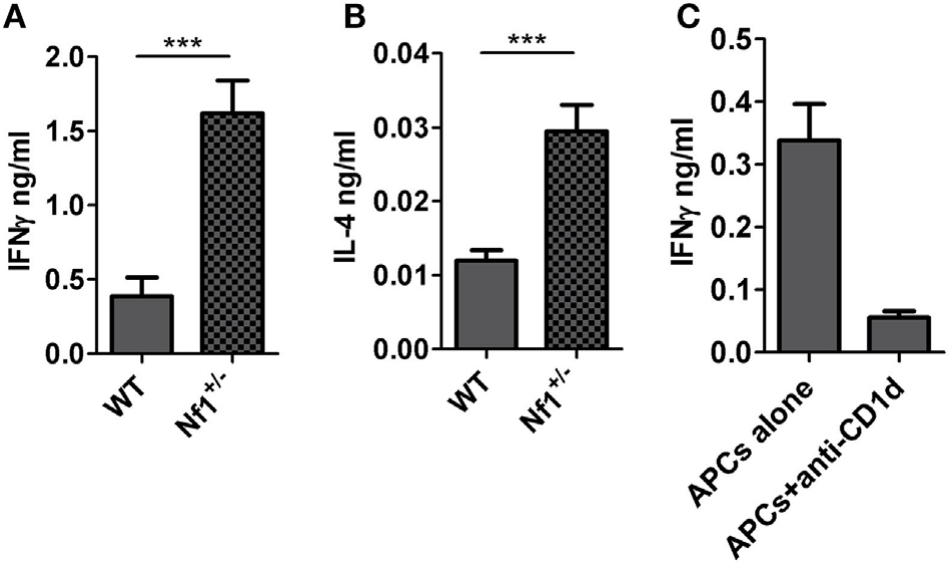
Increased activation of liver *i*NKT cells from *Nf1*^+/−^ mice. Liver mononuclear cells (LMNCs) from individual *Nf1*^+/−^ mice or wildtype (WT) littermates were cocultured with MC57G–mCD1d cells in the presence of α-GalCer for 48 h. Activation of *i*NKT cells was measured by IFN-γ **(A)** and IL-4 **(B)** production into the supernatants. ***p < 0.001. **(C)** MC57G–mCD1d cells were cocultured with LMNCs from WT mice in the presence or absence of the murine CD1d-specific antibody, 1H6, for 48 h. Production of IFN-γ into the supernatants was measured by ELISA. The data are shown as the mean ± SEM. The results are representative of three independent experiments.

Because there was decreased CD1d expression on APCs from *Nf1*^+/−^ mice, we next wanted to find out whether NKT cells from *Nf1*^+/−^ mice were functionally normal *in vitro* and *in vivo*. To test *i*NKT-cell function *in vitro*, LMNCs were cocultured with CD1d-expressing MC57G cells (derived from histocompatible H-2^b^ mice) in the presence of the *i*NKT-cell ligand, α-GalCer. LMNCs from *Nf1*^+/−^ mice were more responsive to CD1d-mediated antigen presentation than those from their WT littermates (Figures 4A,B). The addition of an anti-CD1d antibody blocked the activation of *i*NKT cells (Figure 4C), demonstrating that the NKT-cell activation was CD1d-specific. In contrast to antigen-specific activation, when LMNCs from *Nf1*^+/−^ mice were stimulated with anti-CD3 and anti-CD28 antibodies, they secreted a similar level of cytokines as their WT littermates (Figure S2 in Supplementary Material). Thus, these data demonstrate that *i*NKT cells (but not conventional T cells) from *Nf1*^+/−^ mice are more activated than those from WT littermates upon exogenous lipid Ag stimulation *in vitro*.

To determine whether the NF1 haploinsufficiency would affect NKT-cell function *in vivo*, we injected the *i*NKT-cell ligand α-GalCer to *Nf1*^+/−^ mice and their WT littermates. At different time points, sera were harvested and circulating IL-4 and IFN-γ were measured. *Nf1*^+/−^ mice produced similar levels of these cytokines as their WT littermates (Figure S3 in Supplementary Material). Therefore, these results suggest that *i*NKT cells are functionally normal in *Nf1*^+/−^ mice.

### *Nf1*^+/−^ Mice Bearing RMA/S Tumors Surviving Longer Than WT Mice

Because we observed decreased CD1d expression but increased *i*NKT-cell activity *in vitro* in *Nf1*^+/−^ mice, it was important to determine what the impact of a haploinsufficiency of NF1 would be on CD1d-dependent antitumor activity. Previous reports have suggested that *Nf1*^+/−^ mice are predisposed to developing multiple cancers after 1 year of age, and thus have a shorter life span compared with WT mice (1). To address this question, we inoculated *Nf1*^+/−^ mice and their WT littermates with the RMA/S T cell lymphoma transfected with an empty vector or the murine *cd1d1* cDNA (23). They were then observed for tumor incidence and survival rate. Surprisingly, *Nf1*^+/−^ mice had a better survival rate and longer median survival time (MST) than their WT littermates when they were challenged with either CD1d-positive or CD1d-negative RMA/S tumor cells although, in this experiment, the difference between *Nf1*^+/−^ and WT mice was not statistically significant (Figure S4 in Supplementary Material). Thus, in terms of survival, the antitumor activity in *Nf1*^+/−^ mice exceeds that of their WT littermates.

### Blocking CD1d *In Vivo* Enhancing Antitumor Activity in WT But Not *Nf1*^+/−^ Mice

**Figure 5:**
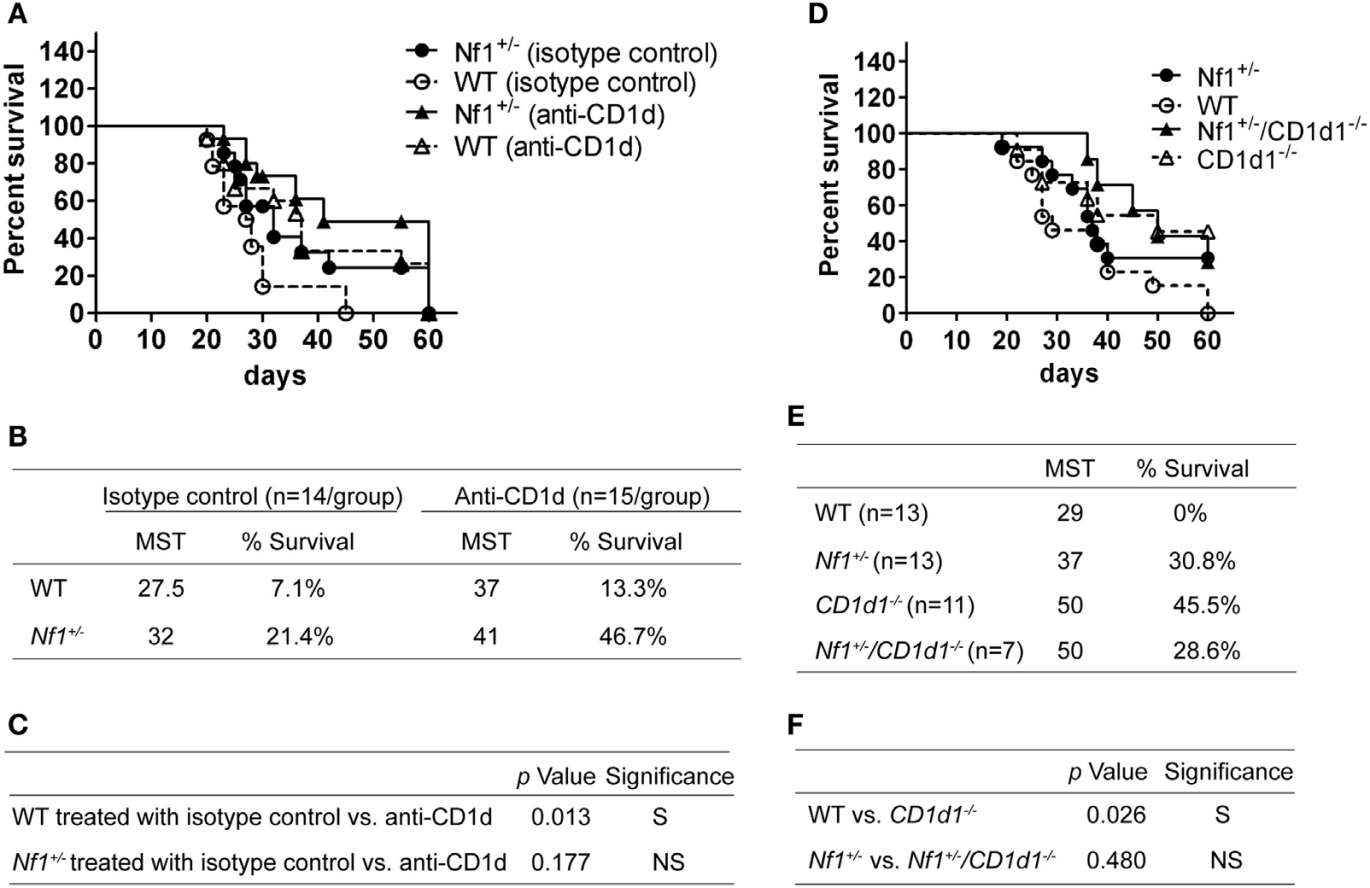
Blocking CD1d *in vivo* enhancing antitumor activity in wildtype (WT) (but not *Nf1*^+/−^) mice. **(A)** *Nf1*^+/−^ mice (black symbols) and their WT littermates (white symbols) were treated i.p. with 50 µg of anti-CD1d antibody 1H6 (triangles) or isotype control (circles) on day 1, and days 5, 10, and 20 posttumor inoculation. The mice were inoculated i.p. with 5 × 10^5^ RMA/S-CD1d cells on day 0 and survival was monitored for up to 60 days posttumor inoculation. Pooled data from three independent experiments are shown. *N* = 14–15 per group. The median survival time (MST) and percent survival on the final day were determined and summarized in **(B)**. Statistical analyses of the survival curves between the different groups are shown in **(C)**. The *p*-values were based on a log-rank test comparing the survival curves of the indicated two groups of mice. S, significant, *p* < 0.05; NS, not significant, *p* > 0.05. **(D)** *Nf1*^+/−^ (black circles), *Nf1*^+/−^/*CD1d1^−/−^* (black triangles) *CD1d1^−/−^* (white triangles), and their WT littermates (white circles) were inoculated with 5 × 10^5^ RMA/S-CD1d cells on day 0 and their survival was monitored for up to 60 days posttumor inoculation. Pooled data from three independent experiments are shown. *N* = 7–13 per group. The MST and percent survival on the final day were determined and summarized in **(E)**. Statistical analyses of the survival curves between the different groups are shown in **(F)**. The *p*-values were based on a Log-rank test comparing the survival curve of the indicated two groups of mice. S, significant, *p* < 0.05; NS, not significant, *p* > 0.05.

Our previous studies have shown enhanced survival in CD1d-deficient mice when they were inoculated with RMA/S T-cell lymphoma cells (23). It was possible that the reduced CD1d expression in *Nf1*^+/−^ mice altered host antitumor activity. To test this hypothesis, *Nf1*^+/−^ and WT mice were treated with an anti-CD1d antibody or isotype control at various times before and after they were inoculated with RMA/S-CD1d cells. Blocking CD1d expression by a CD1d-specific antibody significantly enhanced antitumor activity in WT mice. The CD1d-specific antibody treatment in *Nf1*^+/−^ mice also increased the survival rate of tumor-bearing mice. Although reproducible, the difference was not statistically significant in this experiment (Figures 5A–C). In a parallel experiment, CD1d was also genetically deleted from *Nf1*^+/−^ mice by back-crossing *Nf1*^+/−^ mice to *CD1d1^−/−^* mice. Thus, *Nf1*^+/−^, *CD1d1^−/−^, Nf1*^+/−^*/CD1d1^−/−^*, and WT mice were inoculated with RMA/S-CD1d cells, WT mice had the lowest survival rate among these four different strains of mice (Figures 5D,E). As was observed when CD1d was blocked by antibody *in vivo*, deleting CD1d genetically from WT (but not *Nf1^+/−^*) mice significantly enhanced their survival rate (Figure 5F). Therefore, reduced CD1d expression in *Nf1*^+/−^ mice very likely contributes to host antitumor activity in this model system.

### NF1-Haploinsufficient Type-I (But Not Type II) NKT Cells Suppressing Antitumor Immunity *In Vivo*

**Figure 6:**
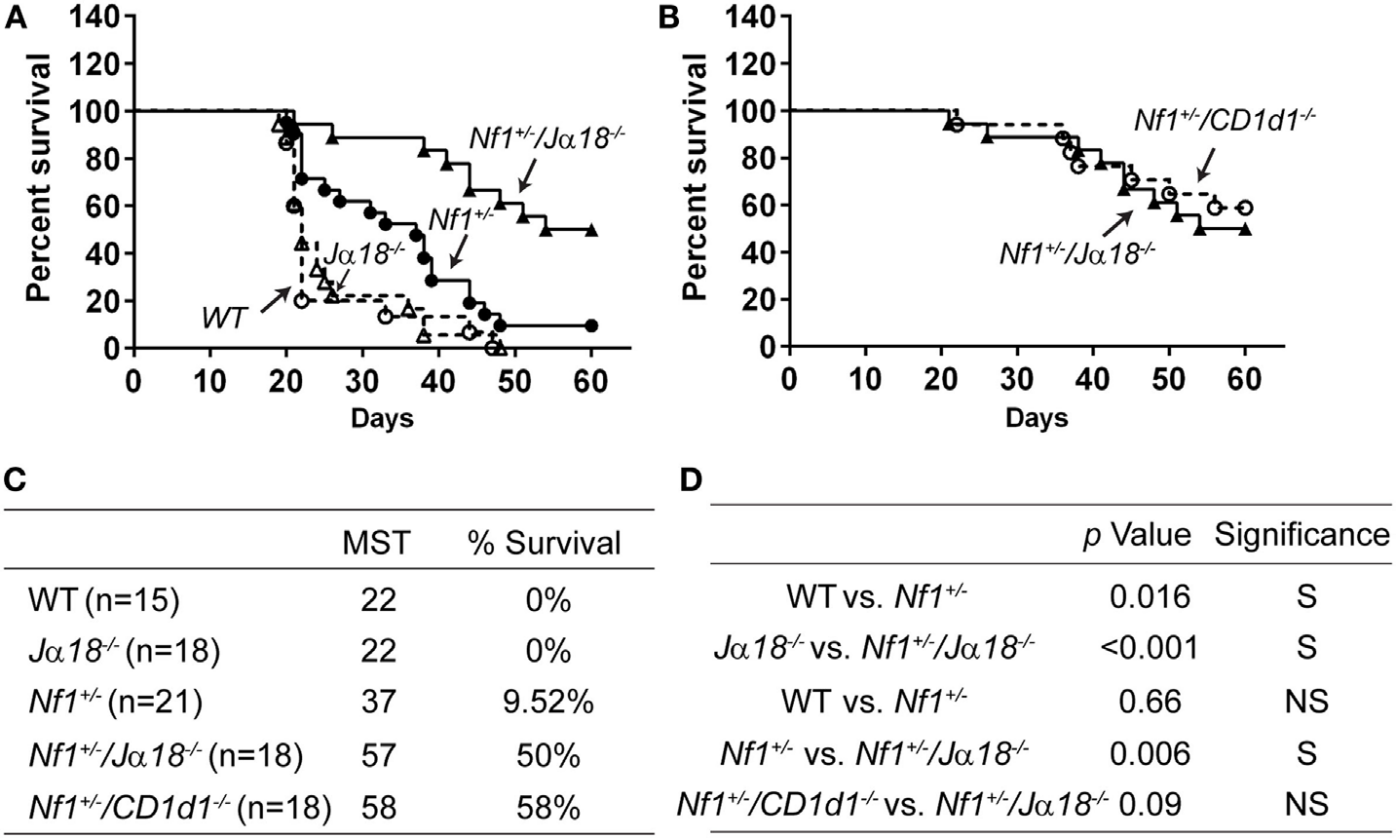
NF1-haploinsufficient Type-I (but not Type-II) NKT cells suppressing antitumor immunity *in vivo*. **(A)** *Nf1*^+/−^ (black circles), *Nf1*^+/−^/*J*α*18^−/−^* (black triangles), *J*α*18^−/−^* (white triangles), and WT littermates (white circles) were inoculated with 5 × 10^5^ RMA/S-CD1d cells on day 0 and survival was monitored for up to 60 days posttumor inoculation. Pooled data from two independent experiments are shown. *N* = 15–21 per group. **(B)** *Nf1*^+/−^/*J*α*18^−/−^* and *Nf1*^+/−^/*CD1d1^−/−^* mice were inoculated with RMA/S-CD1d cells and their survival was monitored as shown in **(A)**. Pooled data from two independent experiments are shown. *N* =18 per group. The MST and percent survival on the final day were determined and summarized in **(C)**. Statistical analyses of the survival curves between the different groups are shown in **(D)**. The *p*-values were based on a Log-rank test comparing the survival curve of the indicated two groups of mice. S, significant, *p* < 0.05; NS, not significant, *p* > 0.05.

CD1d-deficient mice lack both Type-I and Type-II NKT cells, whereas *J*α*18^−/−^* mice only have Type-II NKT cells (29). To determine the impact of NF1 on the antitumor activity of Type-I and Type-II NKT cells, *Nf1*^+/−^ mice were crossed with *J*α*18^−/−^* mice to generate *Nf1*^+/−^/*J*α*18^−/−^* mice. These mice, together with their WT, *Nf1*^+/−^ and *J*α*18^−/−^* littermates were inoculated with RMA/S-CD1d cells. While WT and *J*α*18^−/−^* mice died at a similar rate, as we observed in multiple experiments, *Nf1*^+/−^ mice survived significantly longer than their WT littermates (Figures 6A,C,D). Interestingly, *Nf1*^+/−^/*J*α*18^−/−^* mice had the highest survival rate among the four experimental groups (Figures 6A,C). Thus, the deletion of Type-I NKT cells in *Nf1*^+/−^ mice significantly enhanced survival (Figure 6D), which suggests NF1-haploinsufficent Type-I NKT cells may actually impair antitumor activity. However, as *Nf1*^+/−^/*J*α*18^−/−^* mice survived much longer than their *J*α*18^−/−^* littermates, this would indicate that a haploinsufficiency of NF1 also results in a reduction of the immunosuppressive activity of Type-II NKT cells. In fact, *Nf1*^+/−^/*J*α*18^−/−^* and *Nf1*^+/−^/*CD1d1^−/−^* mice had similar survival rates posttumor inoculation (Figures 6B–D); this suggests that NF1-haploinsufficent Type-II NKT cells in *Nf1*^+/−^/*J*α*18^−/−^* mice are less able to suppress antitumor immunity as compared with WT.

## Discussion

Neurofibromatosis type 1 is a disease caused by mutations in the *NF1* gene, a negative regulator of the Ras signaling pathway. Elevated Ras/ERK activation has been reported in *Nf1*^+/−^ mice and reconstituted *NF1^−/−^* mice, as well as cells from NF1 patients (7, 30). Hyperactivation of the Ras/ERK pathway was also confirmed in the current study by the detection of increased phospho-ERK in the thymus and spleen of *Nf1*^+/−^ mice. Although hyperactivation of the Ras/ERK pathway has been reported to be associated with a defect in NKT-cell development (31), we did not observe any defect in NKT-cell development in *Nf1*^+/−^ mice. Instead, our work has demonstrated that NKT cells from *Nf1*^+/−^ mice actually have enhanced CD1d-dependent activation, compared with those from their WT littermates. We observed similar levels of circulating cytokines in *Nf1*^+/−^ mice after *in vivo* treatment with α-GalCer, even though APCs from *Nf1*^+/−^ mice expressed lower levels of CD1d compared with their WT littermates. This may be explained by the increased responsiveness of NKT cells *in vitro*.

On the other hand, the reduced CD1d expression found in BMDCs from *Nf1*^+/−^ mice suggests that NF1 positively regulates CD1d expression. It is worthwhile to point out that this effect might be due to *in vitro* cultures, as we did not observe a difference in CD1d expression in splenic DCs. It is well-known that NF1 is a negative regulator of the Ras/MAPK pathway (1). We not only observed hyperactivation of the Ras/ERK pathway but also detected elevated Ras/JNK pathway activation in the thymus and spleen of *Nf1*^+/−^ mice. Daginakatte et al. (32) also reported increased JNK activation in *Nf1*^+/−^ microglia cells, but not *Nf1^−/−^* astrocytes, which likely contributed to the increased proliferation of *Nf1*^+/−^ microglia cells in that study. Consistent with our findings, they also did not observe increased p38 activation in these tissues (32). Our results are particularly interesting because we found that blocking the JNK pathway increases (and activation of JNK decreases) CD1d-mediated antigen presentation (Liu et al., manuscript in preparation). In contrast, we previously reported that elevated ERK activation enhances CD1d-mediated antigen presentation during a viral infection by regulating intracellular CD1d trafficking (15). Thus, it is very likely that the reduction of CD1d expression in *Nf1*^+/−^ APCs is an outcome of the combined regulation of multiple signaling pathways as a consequence of the NF1 haploinsufficiency. It is worthwhile to mention that we did not observe any difference in CD1d recycling (Figure S5 in Supplementary Material) or CD1d distribution by confocal microscopy between BMDCs from *Nf1*^+/−^ or WT littermates (data not shown). This suggests that the reduced CD1d expression on the cell surface of *Nf1*^+/−^ APCs is not due to modified CD1d intracellular trafficking caused by an NF1 haploinsufficiency.

Marginal zone B cells, which express a high level of CD1d, are reduced in *Nf1*^+/−^ mice. Moreover, it is known that Notch2 is indispensable to MZB development (33). A recent report suggests that Notch is the effector of NF1 in neurological tissue (34). Thus, a haploinsufficiency of NF1 may affect Notch2 and thereby alter MZB development. However, NF1 may have other unknown functions, not just as a Ras-GAP. A 2,839-amino-acid protein, NF1 contains two major functional domains: a Ras-GAP-related domain (Ras-GRD) and a Sec14-interactive domain. The Sec14-interactive domain is involved in forming a bipartite lipid-binding module and possibly binds to a cellular glycerophospholipid ligand (11). Further investigations are needed to determine how NF1 regulates CD1d expression.

**Figure 7:**
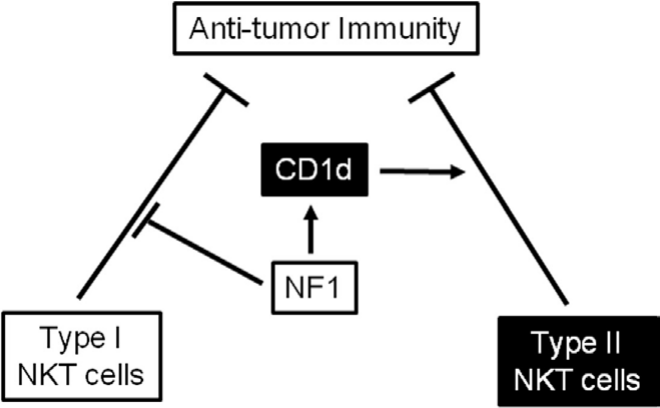
A working model illustrating that neurofibromin 1 (NF1) plays distinct roles in regulating the antitumor activity of Type-I and Type-II NKT cells *in vivo*. NF1 reduces the immunosuppressive activity of Type-I NKT cells, as NF1-haploinsufficient Type-I NKT cells suppress antitumor immunity. In contrast, NF1 expression upregulates CD1d levels and enhances the immunosuppressive activity of Type-II NKT cells. In line with this, a haploinsufficiency in NF1 causes a reduction in CD1d expression and decreases the immunosuppressive activity of Type-II NKT cells, augmenting antitumor immunity.

We observed increased antitumor activity in *Nf1*^+/−^ mice compared with their WT littermates. Although the differences were not always statistically significant, *Nf1*^+/−^ mice consistently survived longer than their WT littermates. Treatment with a CD1d-specific mAb has shown to protect mice from tumor metastasis by several groups (22, 35, 36). It has been suggested that the CD1d mAb may block the activation of “immunosuppressive” Type-II NKT cells (22). Crosslinking CD1d by a specific mAb can also activate antigen-presenting cells, such as DCs, to produce the proinflammatory cytokines IL-12 and IFN-γ (35, 36). Reduced CD1d expression in *Nf1*^+/−^ mice may be somewhat like CD1d-deficient mice (or CD1d mAb-treated mice), in that there is a dysfunction in (or reduced activity of) “immunosuppressive” Type-I NKT cells; this could explain the increased antitumor activity observed in *Nf1*^+/−^ mice. It is also consistent with the findings that blocking CD1d *in vivo* enhanced antitumor activity in WT but *Nf1*^+/−^ mice. In the current study, *WT* and *J*α*18^−/−^* mice died at similar rates and *CD1d1^−/−^* mice survived longer than WT mice. The results suggest that Type-I NKT cells have little impact on antitumor activity whereas Type-I NKT cells are *immunosuppressive* in this model system (Figure 7). *Nf1*^+/−^/*J*α*18^−/−^* mice survived much longer than *Nf1*^+/−^ littermates, suggesting that NF1-haploinsufficient Type-I NKT cells, although demonstrating increased activity *in vitro*, suppressed antitumor activity *in vivo*.

Type-I NKT cells can directly destroy tumor cells, especially those expressing CD1d on their surface, by performing cytolysis *via* perforin, granzyme B, Fas ligand (FasL), and TRAIL (37). Type-I NKT cells can also suppress the function of myeloid-derived suppressor cells (MDSC) and suppressive IL-10-producing neutrophils, to enhance antitumor immunity (20). Type-I NKT cells are capable of producing both Th1 and Th2 cytokines (38). The avidity and stability of antigen/TCR complex determines the type of cytokine production. Strong antigen/TCR interaction causes NKT cells to produce Th1 cytokines, whereas weak antigen/TCR interaction results in Th2 cytokines from NKT cells (39). Th1-biased and IFNγ-producing Type-I NKT cells greatly boost antitumor immunity (20). On the other hand, Type-I NKT cells have been reported to be immunosuppressive by supporting Tregs and/or suppressing tumor-specific CD8^+^ T cells (40, 41). In the current study, the reduced CD1d expression observed in *Nf1*^+/−^ mice may further cause Type-I NKT cells to become Th2-biased and thereby suppress the antitumor activity of CTL and NK cells. In conclusion, NF1-haploinsufficient Type-I NKT cells are more immunosuppressive compared with WT Type-I NKT cells, through a currently unknown mechanism.

In contrast, *Nf1*^+/−^/*J*α*18^−/−^* mice survived much longer than their *J*α*18^−/−^* littermates, indicating that Type-II NKT cells in *Nf1*^+/−^ mice are not as immunosuppressive as WT Type-II NKT cells. *Nf1*^+/−^/*J*α*18^−/−^* mice survived at a similar rate as *Nf1*^+/−^/*CD1^−/−^* mice, further confirming that Type-II NKT cells in *Nf1*^+/−^ mice are not immunosuppressive. Our work suggests that NF1 is required for the immunosuppressive activity of Type-II NKT cells. The reduced CD1d expression in *Nf1*^+/−^ mice may functionally alter Type-II NKT cells, moving from suppressing to enhancing antitumor activity.

A recent publication has suggested that Tregs are also important in the balance of antitumor activity involving Type-I/Type-II NKT cells (29). We did not observe any changes in Tregs in our studies (data not shown). Further studies are needed to investigate tumor immunosurveillance by Type-I/Type-II NKT cells as well as Tregs in *Nf1*^+/−^ mice.

One question raised from our tumor challenge study is the identity of the effector cells that are responsible for removing the tumor cells *in vivo*. Because the RMA/S cell line is Tap-2 deficient, they express a very low level of MHC I molecules on their surface (42). Thus, the effector cells for eliminating RMA/S cells are unlikely to be CD8^+^ cytotoxic T cells. NKT cells have also been shown to exhibit cytotoxicity activity against CD1d^+^ cells (23). However, because *Nf1*^+/−^ mice are more resistant to both CD1d^+^ and CD1d^−^ RMA/S cells, it is unlikely that the sole effector cells are NKT cells. Another population of cytotoxic cells that could play a role here are NK cells. RMA/S cells are highly susceptible to NK cell-mediated lysis (43). We speculate that Type-I and Type-II NKT cells may impact the function of NK cells in the RMA/S tumor model (Figure 7). On the other hand, the cytolytic activity of NK cells is also regulated by many signaling pathways (44). It has been reported that NK cells from vav-1 (a GEF)-deficient mice have reduced cytotoxicity (45), suggesting that NK cell activity may be impacted by changes in Ras/MAPK pathways. However, in the current study, we did not observe increased cytotoxicity by NK cells in *Nf1*^+/−^ mice (Figure S6 in Supplementary Material). Of further interest, a recent report suggests that the inoculation of mice with RMA/S cells causes NK cell anergy and escape from antitumor immunity (46). Importantly, NK cell anergy only occurs in the tumor proximal environment and is likely due to impaired ERK activation downstream of activating receptors on NK cells (46). It is possible that NK cells in *Nf1*^+/−^ mice may be compensated for by reduced ERK phosphorylation and rescue of MAPK/ERK signaling in the tumor microenvironment; thus, *Nf1*^+/−^ mice would exhibit an increase in antitumor immunity and have enhanced survival. Further studies will be focused on how NF1 regulates the antitumor activity of NKT and NK cells in *Nf1*^+/−^ mice.

Neurofibromas are derived from a broad range of cells, including hyperproliferative Schwann cells, fibroblasts, mast cells and perineural cells (47). Loss of heterogyzosity of NF1 in Schwann cells and a heterozygous NF1 microenvironment are both important for the formation of neurofibromas (3). Schwann cells have been shown to express CD1d and can activate NKT cells to secrete anti-inflammatory cytokines (48). We speculate that the absence of NF1 in Schwann cells from NF1 patients may cause a deficiency in CD1d expression. It would be interesting to study the tumor immunosurveillance activity of Type-I and Type-II NKT cells within the neurofibroma microenvironment, where Schwann cells are NF1-deficient. Further studies are necessary to understand the role of the CD1d/NKT-cell axis in NF1-dependent disease progression.

In summary, we have found reduced CD1d expression but increased antitumor activity in a haploinsufficiency model of NF1. This is likely due to reduced immunosuppressive activity by Type-II NKT cells, rather than by an increase in antitumor activity by Type-I NKT cells. The results support the hypothesis that NF1 regulates CD1d-mediated NKT-cell activation and consequent antitumor activity (Figure 7). Future work will focus on investigating how NF1 may regulate the antitumor activity of NKT cells. Our study may therefore provide mechanistic support to target NF1 to improve CD1d/NKT-cell-based immunetherapy.

## Ethics Statement

All animal procedures were approved by the Indiana University School of Medicine’s Institutional Animal Care and Use Committee.

## Author Contributions

RB and JL contributed to the concept and design of the paper. JL, RG, MK, and GR contributed to the acquisition of data. JL, RG, and MK contributed to the analysis and interpretation of data. JL and RB contributed to the writing, review, and/or revision of the manuscript. JL, RG, MK, and GR contributed to the administrative, technical, or material support of the study. RB contributed to the supervision of the study.

## Conflict of Interest Statement

The authors declare that the research was conducted in the absence of any commercial or financial relationships that could be construed as a potential conflict of interest.
