# Supplementary material for: Neurofibromin 1 Impairs Natural Killer T-Cell-Dependent Antitumor Immunity against a T-Cell Lymphoma
Source: Front Immunol. 2018 Jan 5;8:1901. doi: 10.3389/fimmu.2017.01901 (PMC5760513; doi:10.3389/fimmu.2017.01901)
Supplement: Supplementary file 1 [file data_sheet_1.pdf]

Supplemental information

In vitro stimulation of T cells

To activate conventional T cells *in vitro,* splenocytes (2×10^5 cells/well) from *Nf1*$^{+/-}$ mice or WT littermates were stimulated with plate-bound anti-CD3 and anti-CD28 antibodies in triplicate wells of a 96-well microtiter plate for 24h. The supernatants were collected for the measurement of IFN-γ and IL-4 production by ELISA.

Cytolytic assay

Target cells (RMA/S-V or RMA/S-CD1d) were labeled with Calcein-AM (Invitrogen, 15 µl/10^6cells/ml) for 30 min in a 37°C water bath. Labeled target cells (10^4 cells/well) were co-cultured with IL-2-activated NK cells at different effector to target cell (E:T) ratios for 2 h. The supernatants were harvested for analysis by a fluorescent plate reader (excitation 490nm/emission 520nm). For spontaneous release, 100 µl of target cells were mixed with 100 µl of media. For the maximum release, 100 µl of target cells were mixed with 100 µl of 1% NP-40. Percent lysis = 100 × (O.D.- $O.D._{\text{spontaneous release}}$) / ($O.D._{\text{maximum release}}$- $O.D._{\text{spontaneous release}}$).

α-GalCer administration *in vivo*

Nf1$^{+/-}$ mice and WT littermates were injected with 0.8 μg/mouse of α-GalCer, i.v. Sera were harvested at the indicated time intervals for the measurement of IFN-γ, IL-4 and IL-13 by ELISA.

Supplemental figure legends

Figure S1: Lower CD1d expression on cells from *Nf1*$^{+/-}$ mice. Bone marrow-derived dendritic cells (BMDCs) from two individual and representative *Nf1*$^{+/-}$ and WT littermate mice were fixed and stained with CD11c-, CD1d-, MHC I- and MHC II-specific antibodies and analyzed by flow cytometry. The histograms are indicated by black (isotype control) and red (antigen-specific staining) lines. The geometric mean for the indicated surface antigen is shown.

Figure S2: Comparable conventional T cell activation in WT and *Nf1*$^{+/-}$ mice. Splenocytes (2×10^5 cells/well) from *Nf1*$^{+/-}$ mice and WT littermates were stimulated with plate-bound anti-CD3 and anti-CD28 antibodies in triplicate in 96-well microtiter plates for 24h. The supernatants were harvested for the analysis of IL-4 and IFN-γ by ELISA. The data are shown as the mean ± SEM. The results are representative of three independent experiments.

Figure S3: Comparable circulating cytokine levels in WT and *Nf1*$^{+/-}$ mice following an α-GalCer injection *in vivo*. *Nf1*$^{+/-}$ mice (black squares) and their WT littermates (white squares) were injected *i.v.* with α-GalCer (0.8 μg/mouse). At the indicated time points, sera were

harvested for the indicated cytokine ELISA analyses. Each symbol represents an individual mouse.

Figure S4. Greater survival in RMA/S-bearing *Nf1* $^{+/-}$ mice than WT littermates. *Nf1*$^{+/-}$ mice and their WT littermates were inoculated i.p. with 5×10^5 RMA/S-V (A) or RMA/S-CD1d (B) cells. The mice were monitored for up to 60 days post-tumor inoculation. Pooled data from three independent experiments are shown. The ~~mean~~ survival time (MST) and percent survival on the final day were determined and summarized in (C). Statistical analyses of the survival curves between *Nf1* $^{+/-}$ and WT mice are shown in (D). The *p* values were based on a Log-rank test, comparing the survival curves of the indicated two groups of mice.

Figure S5: Comparable recycling rates of CD1d molecules in WT and *Nf1*$^{+/-}$ BMDCs. BMDCs from individual *Nf1*$^{+/-}$ mice (white symbols) and their WT littermates (black symbols) were pre-treated with the protein synthesis inhibitor, cycloheximide. The cells were then blocked with an anti-CD1d antibody (1B1) on ice and incubated at 37°C for the indicated time intervals. Newly recycled surface CD1d molecules were labeled with PE-conjugated 1B1 and analyzed by flow cytometry. The ratio of the mean fluorescent intensity (MFI) of CD1d at each time point as compared to unblocked cells (control = 1) was calculated. Representative data from three independent experiments are shown.

Figure S6: NK cells from *Nf1*$^{+/-}$ and WT littermates have comparable cytolytic activity. Target cells (RMA/S-V or RMA/S-CD1d) were labeled with Calcein-AM for 30 min in a 37°C water bath. The labeled target cells were co-cultured with IL-2-activated NK cells from *Nf1*$^{+/-}$ (black

circles) and WT (white circles) littermates at different effector to target cell (E:T) ratios for 2 h. The supernatants were harvested and read on a fluorescent plate reader. Percent killing = 100 × $(O.D.-O.D._{\text{spontaneous release}})/(O.D._{\text{maximum release}}-O.D._{\text{spontaneous release}})$.

Figure S7. Increased activation of ERK and JNK in the spleen and thymus of *Nf1*$^{+/-}$ mice (original images of Fig.1). Splenocytes, thymocytes and bone marrow-derived dendritic cells (BMDC) were treated with or without Phorbol 12-myristate 13-acetate (100 ng/ml) for 30 min. The cells were then lysed and resolved on a 10% SDS-PAGE gel for the detection of phosphorylated and total ERK1/2 (A, B) and JNK1/2 (C, D) expression by Western blot analysis. Black arrows point to bands corresponding ERK1/2 and JNK1/2. Representative data from at least three independent experiments are shown.

Figure S8: Comparable CD1d expression on thymocytes from *Nf1*$^{+/-}$ mice. (A) Thymocytes from *Nf1*$^{+/-}$ mice and their WT littermates were stained with a CD1d-specific antibody and analyzed by flow cytometry. The mean fluorescent intensity (MFI) of CD1d is shown. Each symbol represents an individual mouse (n=5). (B) Thymocytes from *Nf1*$^{+/-}$ mice and WT littermates were co-cultured with the murine NKT cell hybridoma, N38-2C12. The activation of NKT cells by thymocytes from individual WT and *Nf1*$^{+/-}$ mice was determined by measuring IL-2 secretion in the supernatants by ELISA.
