# Supplementary figures and images for: Neurofibromin 1 Impairs Natural Killer T-Cell-Dependent Antitumor Immunity against a T-Cell Lymphoma
Source: Front Immunol. 2018 Jan 5;8:1901. doi: 10.3389/fimmu.2017.01901 (PMC5760513; doi:10.3389/fimmu.2017.01901)

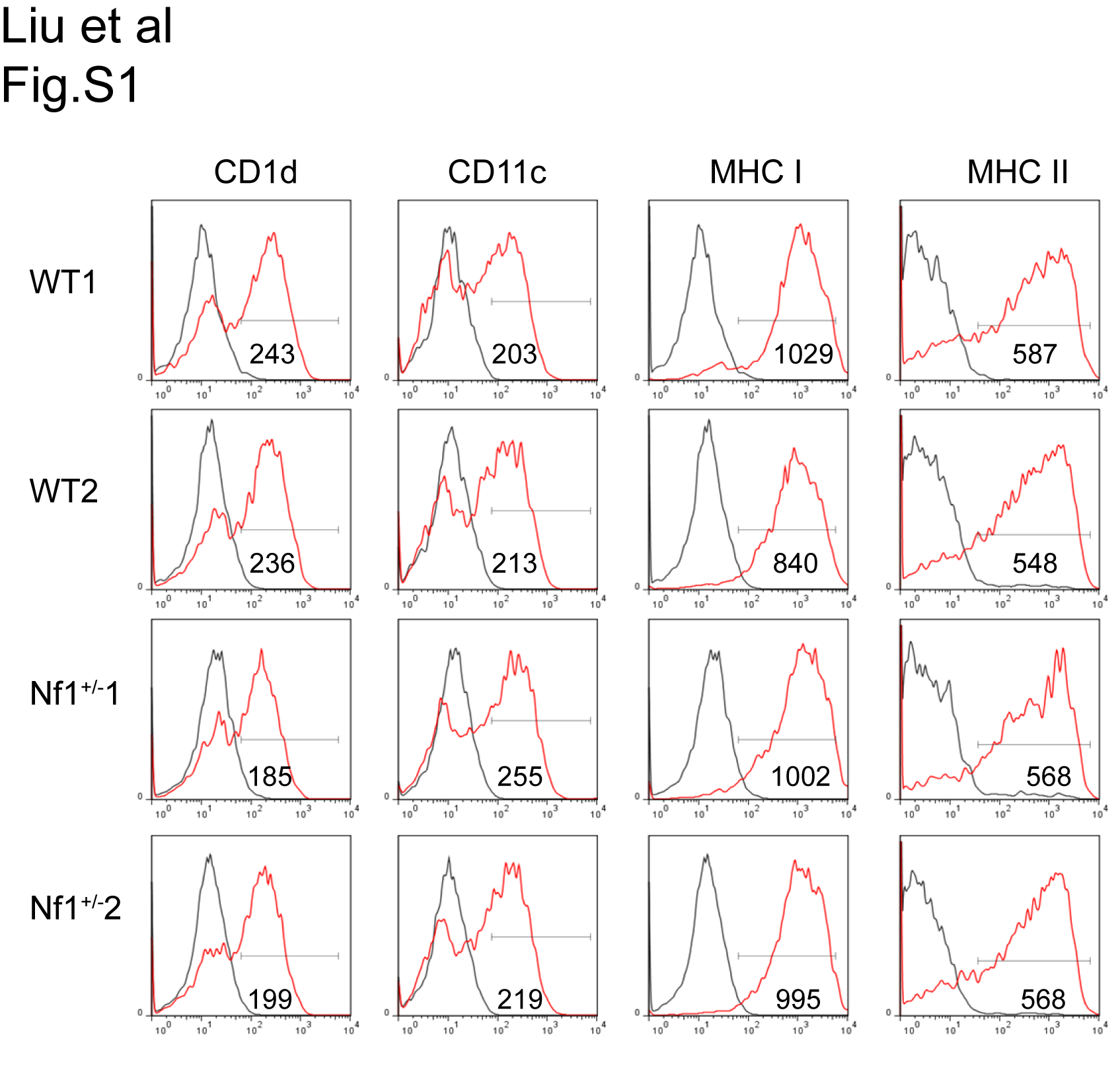

Supplement: Supplementary file 2 [file image_1.tif]

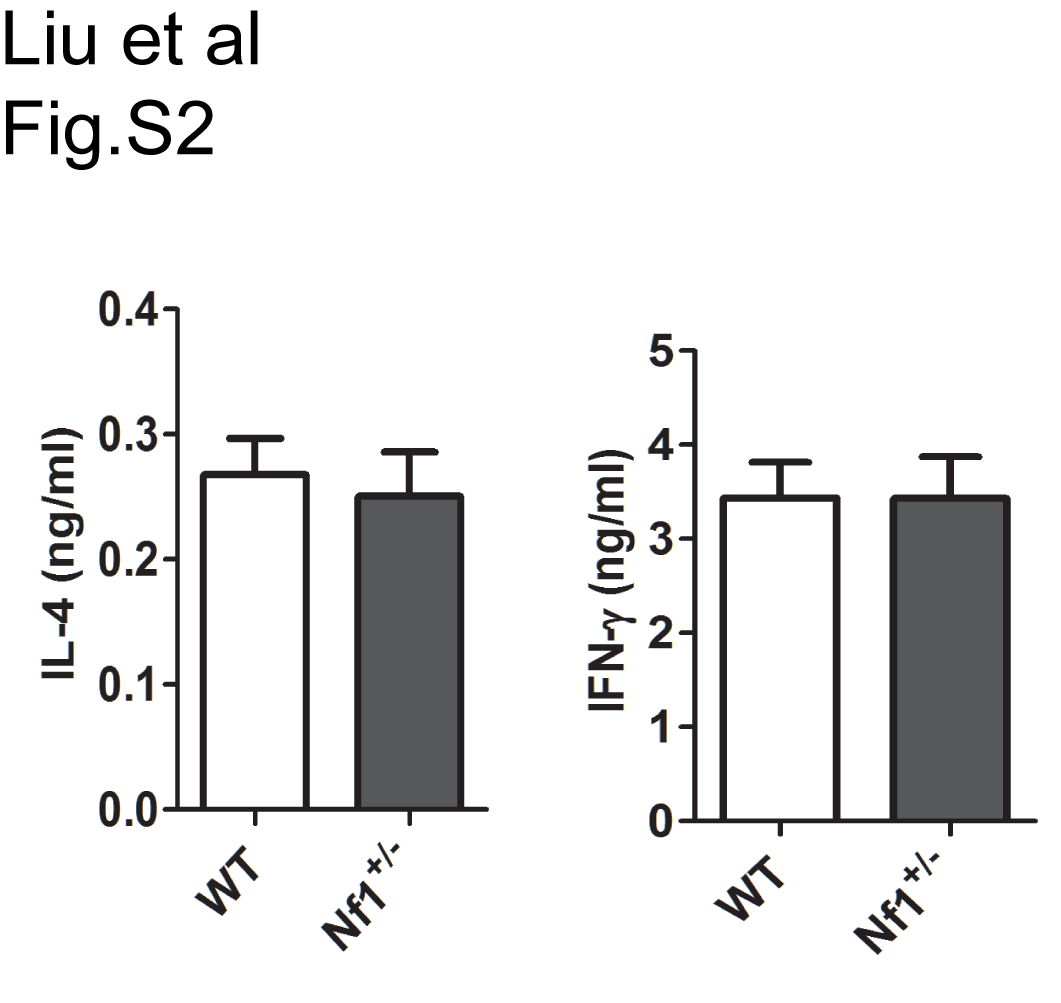

Supplement: Supplementary file 3 [file image_2.tif]

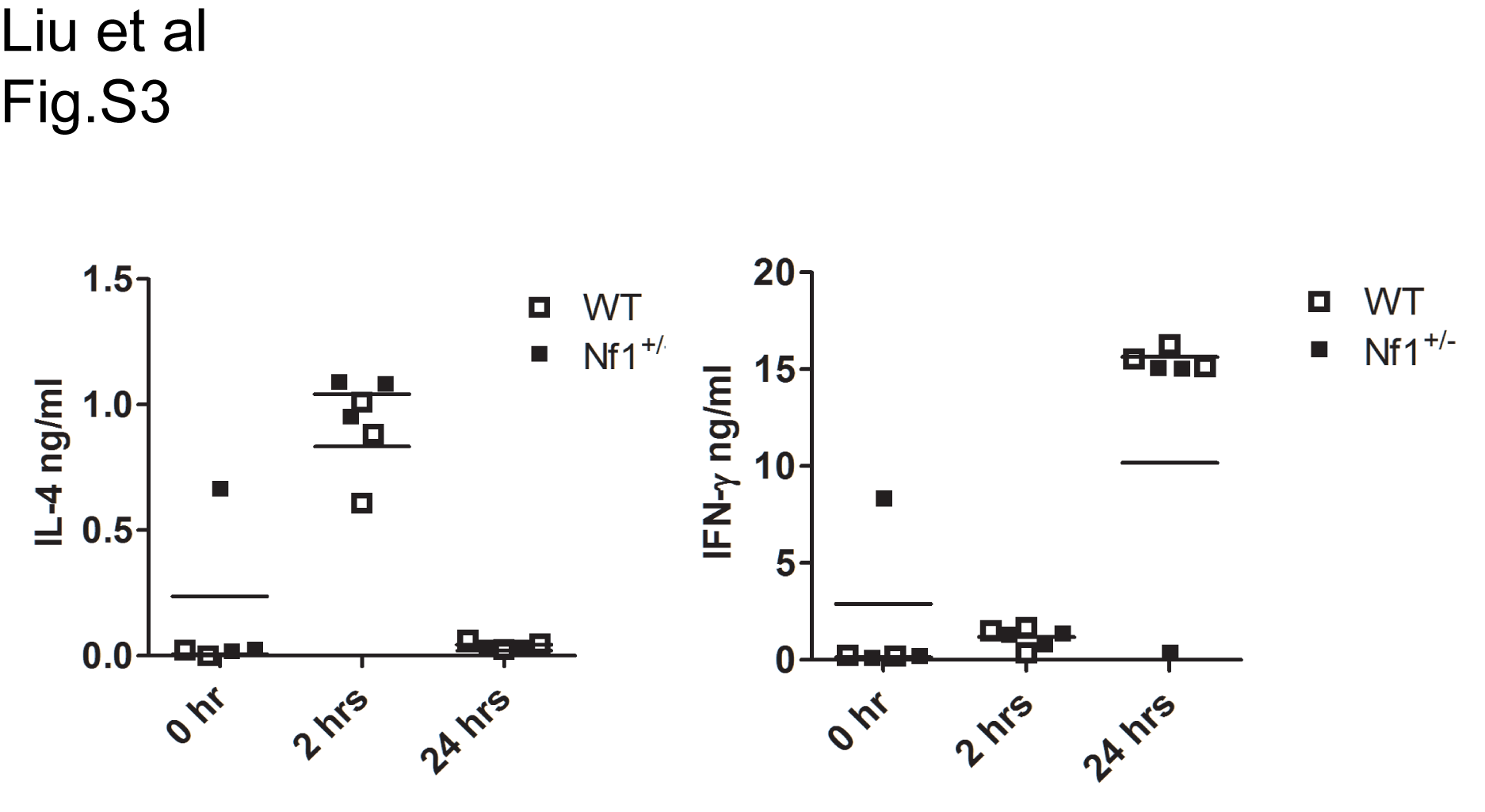

Supplement: Supplementary file 4 [file image_3.tif]

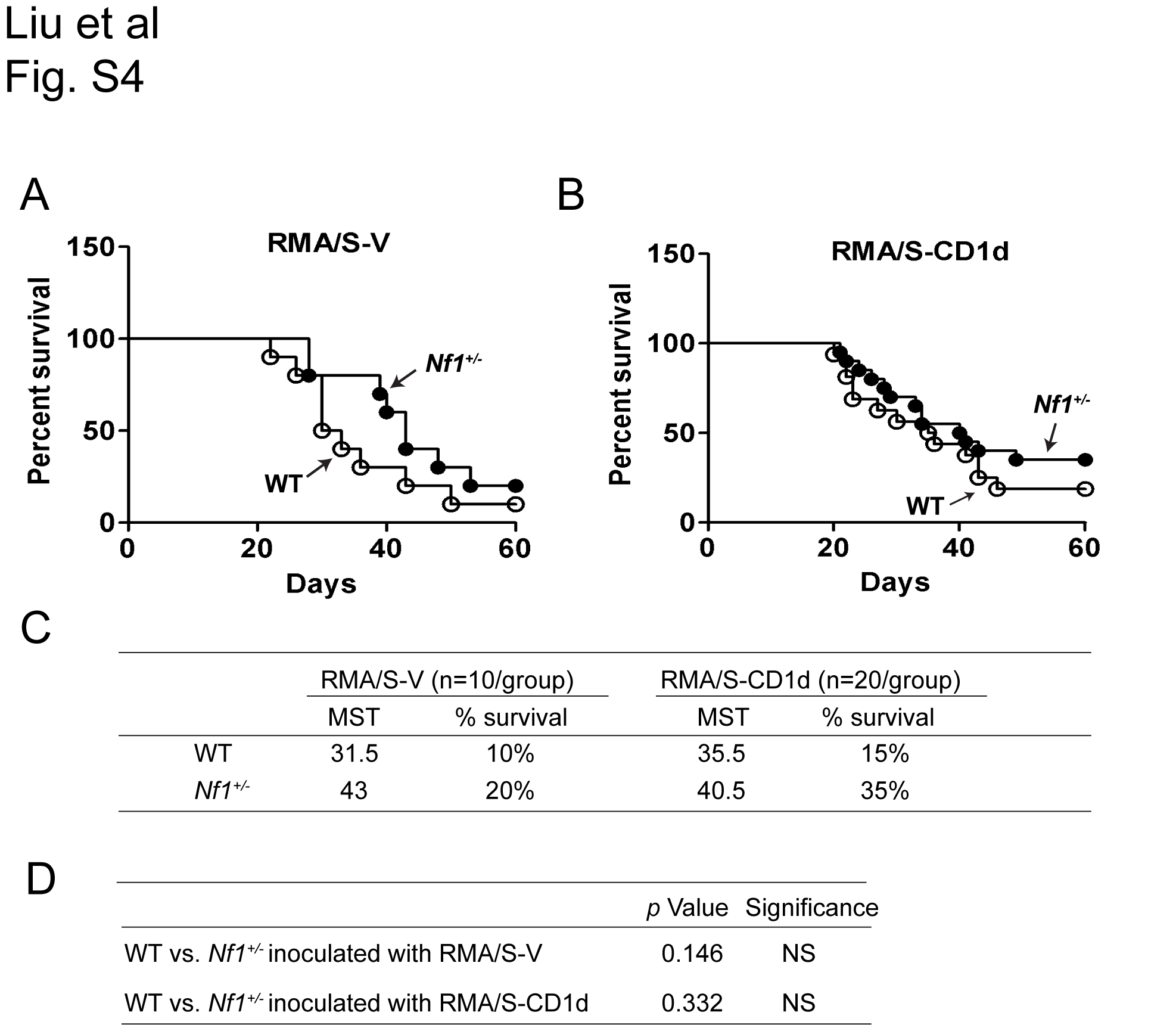

Supplement: Supplementary file 5 [file image_4.tif]

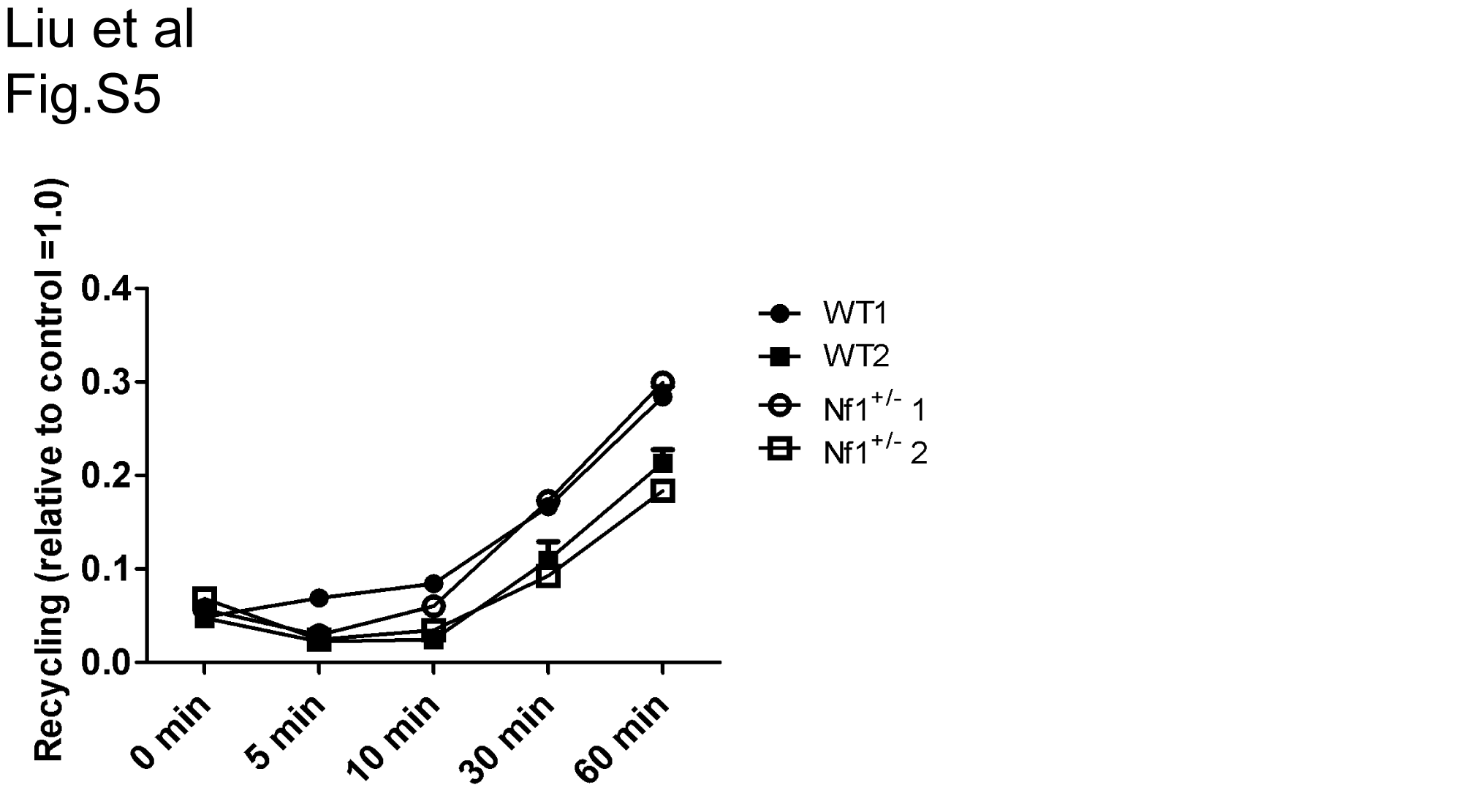

Supplement: Supplementary file 6 [file image_5.tif]

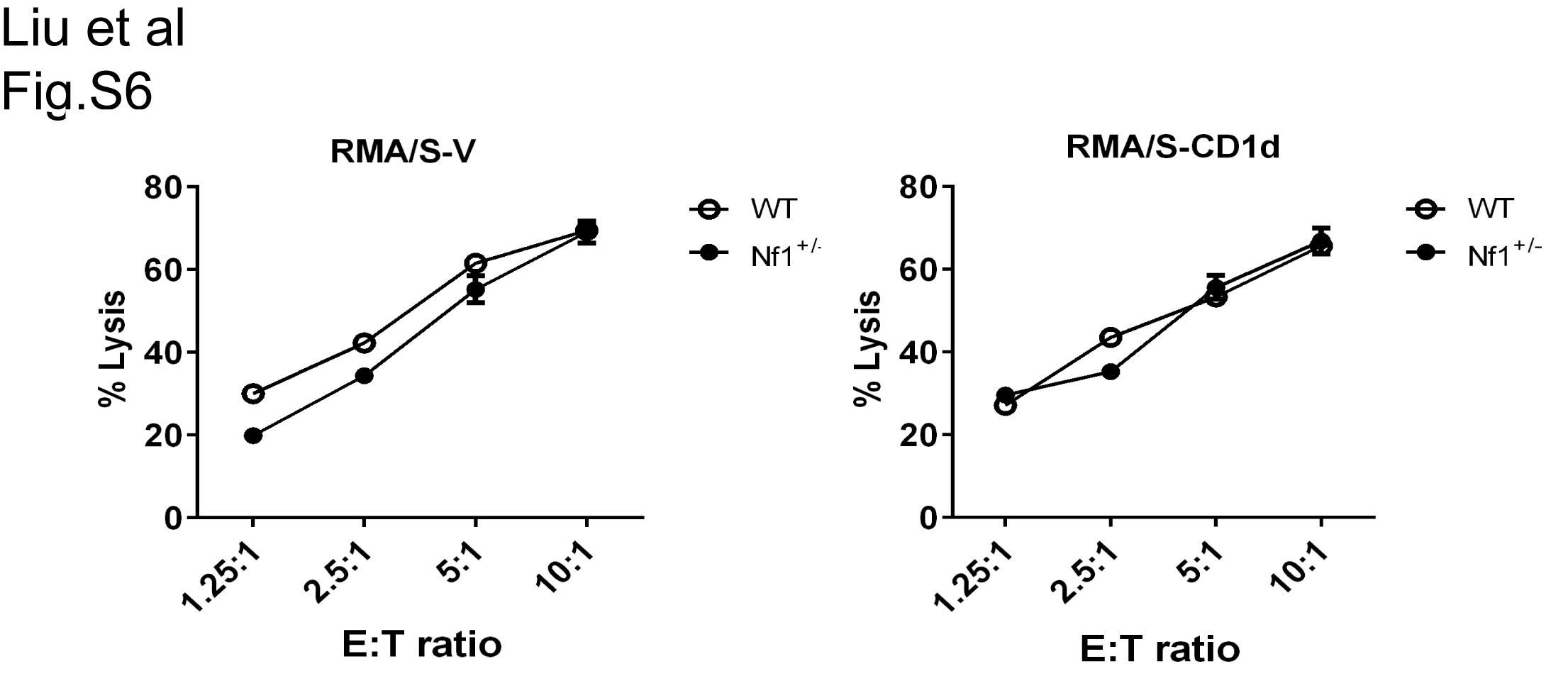

Supplement: Supplementary file 7 [file image_6.tif]

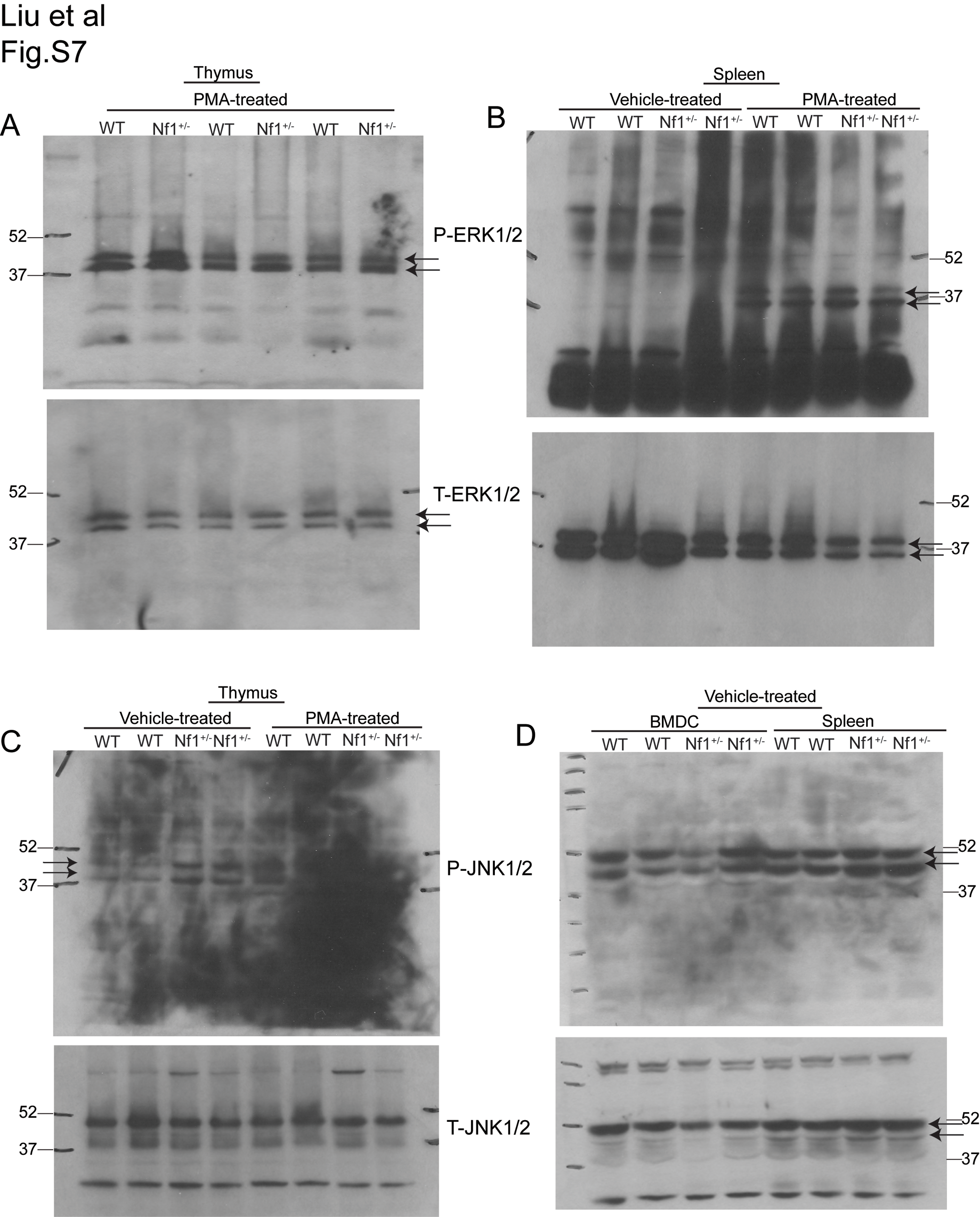

Supplement: Supplementary file 8 [file image_7.tif]

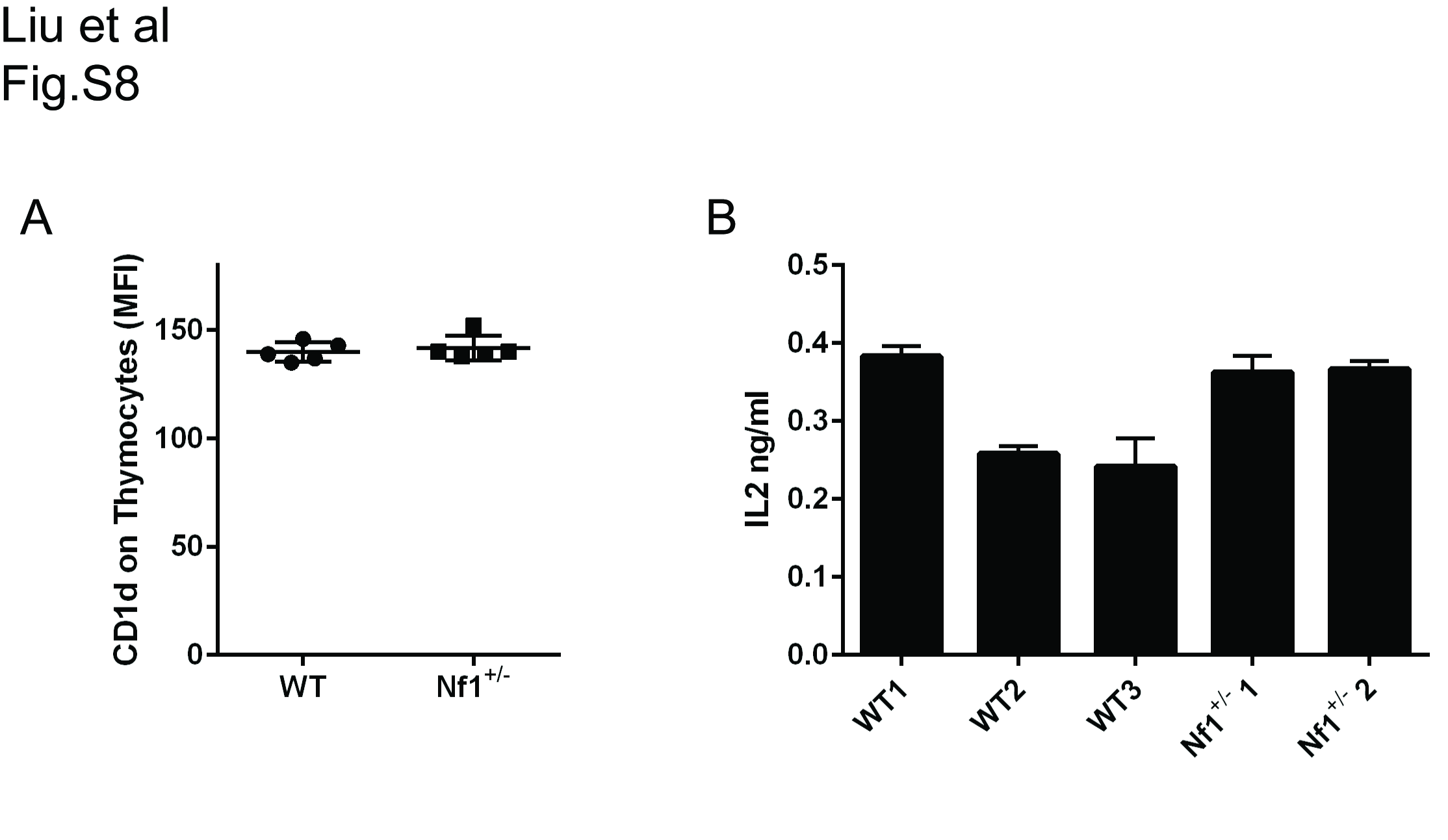

Supplement: Supplementary file 9 [file image_8.tif]
